# Impact of pulmonary hypertension on atrial fibrillation recurrence after pulmonary vein isolation: A prospective multicenter registry study

**DOI:** 10.1016/j.ijcha.2026.101935

**Published:** 2026-05-05

**Authors:** Eias Massalha, Amer Dakka, Ibrahim Marai, Yoav Michowitz, Michael Glikson, Yuval Konstantino, Moti Haim, David Luria, Alexander Omelchenko, Avishag Laish-Farkash, Mahmoud Suleiman, Ariel Furer, Eyal Nof, Roy Beinart, On behalf of the Israeli Working Group on Pacing Electrophysiology

**Affiliations:** a Sheba Medical Center, Tel-Hashomer, Heart Institute, Derech Sheba 2, Ramat Gan, Israel; b Faculty of Medical & Health Sciences, Tel Aviv University, Klatskin St. 35, 6997801 Tel Aviv-Yafo, Israel; c Tzafon Medical Center, Cardiology Department, Poriya, Israel; d The Azrieli Faculty of Medicine in the Galilee, Bar-Ilan University, Safed, Israel; e Jesselson Integrated Heart Center, Shaare Zedek Medical Center, Jerusalem, Israel; f The Hebrew University of Jerusalem, Faculty of Medicine, Jerusalem, Israel; g Department of Cardiology, Cardiac Electrophysiology and Pacing, Soroka Medical Center, Beer-Sheva, Israel; h Ben-Gurion University of the Negev, Beer-Sheva, Israel; i Hadassah Medical Center, Department of Cardiology, Jerusalem, Israel; j Meir Medical Center, Cardiology Department, Kfar Saba, Israel; k Department of Cardiology, Electrophysiology and Pacing Unit, Assuta Ashdod University Medical Center, Ashdod, Israel; l Cardiac Electrophysiology and Pacing, Eyal Ofer Heart Hospital, Rambam Health Care Campus, Haifa, Israel; m The B. Rappaport Faculty of Medicine, Technion, Haifa, Israel; n Department of Military Medicine, Hebrew University of Jerusalem, Faculty of Medicine, Israel

**Keywords:** Atrial fibrillation, Pulmonary hypertension, Catheter ablation, Pulmonary veinisolation, Cryoballoon ablation, Systolic pulmonary artery pressure

## Abstract

**Introduction:**

Pulmonary hypertension (PH) is associated with an increased risk of atrial fibrillation (AF), and AF onset in PH may signal advanced disease. While catheter ablation (CA) offers clinical benefits, post-ablation recurrence remains a challenge. We evaluated whether systolic pulmonary artery pressure (sPAP) predicts AF recurrence following pulmonary vein isolation (PVI).

**Methods:**

Data from the prospective, multicenter Israeli Catheter Ablation Registry (ICAR) included 485 patients undergoing PVI between January 2019 and December 2021, all with echocardiographic sPAP measurements. Patients were stratified into two groups based on sPAP values: high-probability PH (sPAP > 45 mmHg) and low/intermediate probability PH (sPAP ≤ 45 mmHg). AF recurrence within 12 months was assessed, along with subgroup analyses and evaluation of procedural complications.

**Results:**

Patients with high-probability PH were older (69.05 ± 8.75 vs. 64.34 ± 11.27 years; p < 0.01) and had more comorbidities. Cryoballoon ablation was utilized in 387 patients (79.8%). High-probability PH patients had significantly higher 12-month AF recurrence rates (30.4% vs. 17.2%; p = 0.029), despite similar acute procedural success and overall periprocedural complication rates. A Cox proportional hazards model identified sPAP > 45 mmHg as an independent predictor of recurrence (adjusted HR 2.55; p < 0.01), while spline analysis demonstrated a dose-dependent relationship between rising sPAP values and recurrence.

**Conclusion:**

This study emphasizes the importance of PH, evaluated through echocardiographic sPAP values, in predicting post-PVI AF recurrence. These findings support incorporating echocardiographic sPAP into pre-procedural risk assessment to guide customized post-ablation monitoring.

## 1 Introduction

Patients with pulmonary hypertension (PH) have an elevated risk of developing atrial fibrillation (AF) due to persistent right atrial (RA) pressure overload, stretching, and subsequent remodeling [1], [2], [3], [4], [5]. In patients with PH and coexisting AF, the occurrence of AF may indicate a more advanced stage of PH, and is an independent risk factor for mortality [3], [4], [5], [6]. The development of AF in patients with PH is often accompanied by clinical worsening and right heart failure (HF) [4].

Restoring and maintaining sinus rhythm in AF patients with concurrent PH leads to notable clinical benefits, yet managing this group of patients presents substantial challenges [3], [6], [7]. Current guidelines advocate for rhythm control in patients with PH and either AF or atrial flutter (AFL) to enhance functional status and improve survival [1], [8], [9]. Additionally, they emphasize the importance of multidisciplinary care involving cardiologists, pulmonologists, and electrophysiologists with expertise in both conditions [1], [8].

Despite the effectiveness of catheter ablation (CA) in restoring sinus rhythm, AF recurrence remains a significant challenge [2], [3], [8], [10], emphasizing the importance of identifying underlying predictive factors. Preprocedural predictors such as AF type and left atrial (LA) size are well-established [2], [11], [12], [13], [14]. However, evidence on the association between echocardiographically estimated systolic pulmonary artery pressure (sPAP) and AF outcomes remains limited [15], [16], [17], [18], with no large-scale or prospective studies conducted to date.

This study utilizes data from the prospective, multicenter Israeli Catheter Ablation Registry (ICAR) and aims to evaluate the relationship between estimated sPAP and the recurrence rates of AF following CA.

## 2 Methods

### 2.1 Study population & data collection

The Israeli Catheter Ablation Registry (ICAR) is a prospective, multicenter registry encompassing all patients who underwent AF ablation from January 2019 to December 2021 across 14 electrophysiology centers nationwide. This registry is a collaborative effort by the cardiac community and is managed by the Israeli Center for Cardiovascular Research (ICCR). Ethical approval was obtained from the ethics committees of all participating institutions, and patients provided written consent. The registry collects demographic information, clinical characteristics, imaging reports, specific procedural data, and outcomes for each patient. Patients with inadequate echocardiography results or invalid estimated sPAP measurements were excluded.

All data were collected prospectively during the patient's initial hospital admission for ablation. The managing electrophysiologist documented the information into a secure, web-based electronic case report form using REDCap software; protected by a firewall and password. Collected variables included baseline patient characteristics (demographics, comorbidities, vital signs, lab results, and imaging reports); procedural history, and concurrent and prior medical therapy (including antiarrhythmic drugs (AADs), rate control, anticoagulation, and antiplatelet drugs). Procedural specifics included ablation energy source, duration from skin to skin; pulmonary vein anatomy; pulmonary vein occlusion, nadir temperature, time to isolation, fluoroscopy duration, and immediate complications. Echocardiographic indices, such as left ventricular ejection fraction (LVEF), left ventricular end-diastolic diameter (LVEDD), LA diameter, sPAP, left ventricular hypertrophy (LVH), mitral regurgitation (MR), and tricuspid regurgitation (TR), were collected throughout the study.

### 2.2 Echocardiographic sPAP Assesment and ablation procedural approach

Pulmonary hypertension (PH) is characterized by an elevated mean pulmonary artery pressure (mPAP) of 20 mm Hg or more at rest [9]. The definitive diagnostic method for PH is right heart catheterization (RHC) [9], [19], [20], [21]. However, echocardiography offers a safe and readily accessible alternative for estimating sPAP [9], [22]. This non-invasive technique typically employs continuous wave (CW) Doppler to measure the peak velocity of the tricuspid regurgitant (TR) jet, providing an estimate that closely aligns with the sPAP measurements obtained from RHC [19], [22]. Although current guidelines prioritize TRV for PH probability assessment [9], TRV was not consistently available in this cohort, whereas sPAP was routinely recorded and sPAP > 45 mmHg has been widely associated with moderate PH and adverse cardiovascular outcomes [23], [24], accordingly, high-probability PH was defined as sPAP > 45 mmHg. For patients with atrial fibrillation (AF) during an echocardiography study, averaging multiple TR jet velocity readings ensures a more accurate assessment of the pressure difference [19].

In first ablation procedures, pulmonary vein isolation was the primary strategy in both paroxysmal and persistent AF patients, whereas in repeat ablation procedures, electroanatomical mapping systems were commonly used and additional substrate modification, including posterior wall ablation and CFAE targeting, was performed at the discretion of the treating electrophysiologist.

Transseptal puncture was performed under fluoroscopy and/or intracardiac echocardiography guidance with SafeSept assistance, without routine acquisition of invasive cardiac chamber pressure measurements.

### 2.3 Follow-up

Patients received prescriptions for oral anticoagulants for a minimum of two months post-ablation, with the continuation or discontinuation of anticoagulation and antiarrhythmic drugs (AADs) determined by the treating physician. In the absence of documented atrial fibrillation recurrence, AAD therapy was generally discontinued at the routine follow-up visit 3–6 months after ablation, irrespective of pH category. Follow-up care involved directly engaging patients and reviewing their clinical and hospital records upon readmission. Standard follow-up included outpatient visits at 3 to 6 months and then annually or earlier if symptoms suggestive of arrhythmia recurrence were observed. Patients underwent electrocardiograms (ECGs) and 48-hour Holter monitoring during these visits. Documentation covered all occurrences of direct current (DC) cardioversions, repeat procedures, AAD usage, and both acute and chronic complications. Documented complications encompassed tamponade, thromboembolism, neurologic events including stroke and transient ischemic attack, phrenic nerve paralysis, heart block, pericarditis, significant vascular issues requiring medical intervention or prolonged hospital stays, atrio-esophageal fistulas, and mortality.

### 2.4 Outcomes

The primary efficacy endpoint was defined as confirmed AF recurrence lasting at least 30 s, occurring after the eight-week post-procedure blanking period [8]. AF recurrences were documented using either a clinical 12-lead ECG or an ambulatory monitor. Any episodes of AF within the initial eight weeks following ablation were excluded from the assessment of primary clinical recurrence.

The study cohort was divided into two groups based on sPAP measurements: one group with sPAP ≤ 45 mm Hg and another with sPAP > 45 mm Hg. This cutoff was chosen based on the assumption that patients with sPAP > 45 mm Hg have PH with high probability, corresponding to an echocardiographic peak TRV of > 3.4 m/s [9], [23], [24], [25].

### 2.5 Statistical analysis

Statistical analyses were conducted using two-tailed tests, with a significance threshold set at p < 0.05. Variables were summarized based on their characteristics. Categorical variables were presented as frequencies and percentages, with group differences evaluated using the chi-square test or Fisher’s exact test, as appropriate.

The distribution of continuous variables was assessed for normality using the Kolmogorov-Smirnov test, QQ-plot visualization, and analysis of residual distribution and variance. Variables with a normal distribution were expressed as means and standard deviations (SD), and group comparisons were performed using the Student’s *t*-test. For non-normally distributed variables, medians and interquartile ranges (IQR; 25th–75th percentiles) were reported, with significance assessed using the Mann-Whitney *U* test.

The association between clinical and echocardiographic variables and the risk of AF recurrence following CA with PVI was evaluated using Cox proportional hazards regression models. Variables were selected for inclusion in multivariate models based on statistical significance in univariate analyses (p < 0.05), established clinical relevance, and evidence from prior studies supporting their plausibility and potential impact on outcomes. The specific variables included are detailed in Table 4.

Statistical analyses were conducted using SPSS statistical software version 27.0.0 (IBM, Armonk, NY, USA) and R software version 4.3.1 (The R Foundation).

## 3 Results

### 3.1 Baseline characteristics

The final study cohort consisted of 485 patients who underwent pulmonary vein isolation (PVI) from January 2019 to December 2021 and had valid documented sPAP measurements.

The cohort consisted of 485 patients with an average age of 64.9 ± 11.1 years, including 193 (39.8%) females. Paroxysmal AF was present in 294 patients (60.7%) (Table 1). The majority, 387 patients (79.8%), underwent cryoballoon ablation (CBA), while the remaining 98 patients (20.2%) received radiofrequency ablation (RFA) (Table 2). The cohort mean sPAP was 32.69 ± 10.62 mm Hg (Table 2). Based on sPAP measurements, patients were categorized into two groups: 429 patients (88.4%) had a low/intermediate PH probability (sPAP ≤ 45 mm Hg), and 56 patients (11.5%) were classified as having a high-probability PH (sPAP > 45 mm Hg).

**Table 1:** Baseline Characteristics Stratified by Pulmonary Hypertension Severity.

**Characteristic**		**Overall** **(N = 485)**	Low/int. probability PH* (N = 429)	High-probability PH** (N = 56)	***P* value**
**Clinical characteristics**					
	Age, mean ± SD (y)	64.89 ± 11.10	64.34 ± 11.27	69.05 ± 8.75	< 0.01

	Female sex, n (%)	193 (39.8)	168 (39.2)	25 (44.6)	0.52
	BMI, mean ± SD (kg/m^2^)	29.09 ± 5.53	28.95 ± 5.56	30.28 ± 5.18	0.113
	Clinical HF (NYHA 2–4), n (%)	77 (16.1)	53 (12.5)	24 (45.3)	< 0.01
	Ischemic heart disease, n (%)	79 (16.3)	63 (14.7)	16 (28.6)	0.014
	Non-ischemic CM, n (%)	69 (14.2)	57 (13.3)	12 (21.4)	0.151
	Prior myocardial infarction	44 (9.1)	32 (7.5)	12 (21.4)	< 0.01
	Hypertension, n (%)	307 (63.4)	262 (61.2)	45 (80.4)	< 0.01
	Diabetes mellitus, n (%)	124 (25.6)	100 (23.4)	24 (42.9)	< 0.01
	Cerebrovascular disease, n (%)	44 (9.1)	40 (9.3)	4 (7.1)	0.774
	Peripheral vascular disease, n (%)	16 (3.3)	15 (3.5)	1 (1.8)	0.78
	COPD, n (%)	43 (8.9)	31 (7.2)	12 (21.4)	< 0.01
	Renal dysfunction, n (%)	50 (10.3)	39 (9.1)	11 (19.6)	0.027
	Obstructive sleep apnea, n (%)	89 (18.5)	78 (18.3)	11 (20)	0.899
**AF characteristics**					
	Persistent AF, n (%)	191 (39.4)	158 (36.8)	33 (58.9)	< 0.01
	Atrial flutter, n (%)	112 (23.5)	92 (21.9)	20 (36.4)	0.027
	AF duration, mean ± SD (y)	3.96 ± 3.96	3.98 ± 3.97	3.80 ± 3.9	0.759
	Prior cardioversion, n (%)	279 (60.1)	244 (59.4)	35 (66)	0.433
	Prior AF ablation, n (%)	37 (7.7)	33 (7.8)	4 (7.3)	>0.99
	Antiplatelet therapy, n (%)	28 (5.8)	25 (5.8)	3 (5.4)	>0.99
	Rate control medication, n (%)	472 (98.7)	420 (98.8)	52 (98.1)	>0.99
	Pre-procedure AAD, n (%)	315 (64.9)	270 (62.9)	45 (80.4)	0.015
	Failure of one AAD, n (%)	352 (85.2)	317 (85.2)	35 (85.4)	>0.99

Abbreviation: AAD, antiarrhythmic drug; AF, atrial fibrillation; COPD, chronic obstructive pulmonary disease; CM, cardiomyopathy; int., intermediate; IQR, interquartile range; NYHA, New York Heart Association; sPAP, systolic pulmonary artery pressure; PH, pulmonary hypertension; SD, standard deviation; y, years.

* This cohort includes patients with estimated sPAP ≤ 45 mm Hg and those with normal estimated sPAP.

** This cohort includes patients with estimated sPAP > 45 mm Hg.

**Table 2:** Electrocardiographic, Echocardiographic, and Procedural Characteristics.

**Characteristic**			**Overall** **(N = 485)**	Low/int. probability PH* (N = 429)	High-probability PH** (N = 56)	***P* value**
**Echocardiographic & electrocardiographic characteristics**						
	Sinus rhythm at admission, n (%)		294 (60.7)	271 (63.3)	23 (41.1)	< 0.01
	Bundle branch block, n (%)		51 (10.6)	40 (9.4)	11 (20)	0.03
	LVEF, mean ± SD (%)		54.52 ± 10.69	55.35 ± 9.76	48.02 ± 14.82	<0.001
	Left ventricular hypertrophy, n (%)		131 (27)	113 (26.3)	18 (32.1)	0.447
	Left atrial enlargement, n (%)		135 (27.8)	100 (23.3)	35 (62.5)	< 0.01
	Left atrial size, mean ± SD (mm)		42.17 ± 7.34	41.74 ± 7.31	45.61 ± 6.68	< 0.01
	sPAP, mean ± SD (mm Hg)		32.69 ± 10.62	29.92 ± 7.32	53.94 ± 7.55	< 0.01
	Significant mitral regurgitation, n (%)		76 (15.7)	47 (11)	29 (51.8)	< 0.01
	Significant mitral stenosis, n (%)		17 (3.6)	14 (3.3)	3 (5.7)	0.631
	Significant tricuspid regurgitation, n (%)		25 (5.2)	16 (3.7)	9 (16.1)	< 0.01
**Procedural indices**						
	Procedure duration, median [IQR] (min)		90 [60,120]	90 [60,120]	110 [80,180]	< 0.01
	Energy source					0.04
		Cryoballoon ablation, n (%)	387 (79.8)	348 (81.1)	39 (69.6)	

		Radiofrequency ablation, n (%)	98 (20.2)	81 (18.8)	17 (30.3)	

Abbreviation: AF, atrial fibrillation; IQR, interquartile range; sPAP, systolic pulmonary artery pressure; PH, pulmonary hypertension; SD, standard deviation.

* This cohort includes patients with estimated sPAP ≤ 45 mm Hg and those with normal estimated sPAP.

** This cohort includes patients with estimated sPAP > 45 mm Hg.

Patients with high-probability PH were significantly older (69.05 ± 8.75 vs. 64.34 ± 11.27 years; p < 0.01) (Table 1). No significant gender differences were observed between the groups. The high-probability PH group had a markedly higher prevalence of comorbidities, including HF (45.3% vs. 12.5%; p < 0.01), ischemic heart disease (IHD) (28.6% vs. 14.7%; p = 0.01), and hypertension (80.4% vs. 61.2%; p < 0.01). Additionally, this group exhibited increased rates of diabetes mellitus (DM) (42.9% vs. 23.4%; p < 0.01), chronic obstructive pulmonary disease (COPD) (21.4% vs. 7.2%; p < 0.01), and renal dysfunction (19.6% vs. 9.1%; p = 0.02) (Table 1).

Within the high-probability PH group, PH was most attributed to left heart disease (31/56), followed by pulmonary-related etiologies (8/56), mixed cardiac and pulmonary causes (5/56), and other etiologies (12/56).

Regarding AF characteristics, patients with high-probability PH exhibited a higher AF burden, including higher rates of persistent AF (58.9% vs. 36.7%; p < 0.01), as well as concomitant AFL (36.4% vs. 21.9%; p = 0.027). Additionally, these patients were more frequently on pre-procedure antiarrhythmic therapy (80.4% vs. 62.9%; p = 0.015). AF duration, however, did not differ significantly between the groups (p = 0.76) (Table 1).

### 3.2 Echocardiographic indices

Patients with high-probability PH had a notably lower LVEF (48.02 ± 14.82 vs. 55.35 ± 9.76; p < 0.01). Left atrium (LA) enlargement was considerably more prevalent in these patients (62.5% vs. 23.3%; p < 0.01), along with a larger mean LA size (45.61 ± 6.68 mm vs. 41.74 ± 7.31 mm; p < 0.01). Additionally, high-probability PH was associated with significantly higher rates of both significant mitral and tricuspid regurgitation (51.8% vs. 11%; p < 0.01, and 16.1% vs. 3.7%; p < 0.01, respectively) (Table 2).

### 3.3 Procedural indices and complications

RFA was used more commonly in patients with high-probability PH, and the procedure had a longer median duration (110 min vs. 90 min; p < 0.01) (Table 2).

The overall complication rate was relatively low (10.3%), with no statistically significant differences in most complications between patients with low/intermediate versus high-probability PH. Pulmonary complications were rare and comparable between the two groups, with no documented cases of clinically significant right-to-left interatrial shunting. Notably, we observed a higher incidence of neurological events in the high-probability PH group (3.6% vs. 0.2%; p = 0.037), indicating an elevated risk in this cohort (Table 3).

**Table 3:** Procedural Complications and Outcomes Stratified by Pulmonary Hypertension Severity.

			**Overall (N = 485)**	Low/int. probability PH*(n = 429)	High-probability PH**(N = 56)	***P* value**
Complications						
	Phrenic nerve palsy/injury, n (%)		34 (7.0)	31 (7.2)	3 (5.4)	0.813
	Intraprocedural cardioversion, n (%)		157 (32.5)	134 (31.4)	23 (41.1)	0.192
	Peripheral vascular events, n (%)		8 (1.7)	8 (1.9)	0 (0)	0.634
	Neurologic events, n (%)#		3 (0.6)	1 (0.2)	2 (3.6)	0.037
	Pulmonary events, n (%)		2 (0.4)	1 (0.2)	1 (1.8)	0.554
	Combined periprocedural complications, n (%)†		50 (10.3)	45 (10.5)	5 (8.9)	0.72
**Outcomes**						
	Isolation of all pulmonary veins, n (%)		449 (92.6)	399 (93)	50 (89.3)	0.467
	Sinus rhythm at discharge, n (%)		477 (98.4)	421 (98.1)	56 (100)	0.636
	Anticoagulant therapy at discharge, n (%)		473 (97.5)	417 (97.2)	56 (100)	0.418
	Rate control therapy at discharge, n (%)		278 (57.6)	243 (56.9)	35 (62.5)	0.514
	Antiarrhythmic therapy at discharge, n (%)		361 (74.7)	319 (74.7)	42 (75)	0.99
	Deceased within 12 mo., n (%)		3 (0.6)	1 (0.2)	2 (3.6)	0.037
	AF recurrence during blanking period, n (%)		46 (9.5)	36 (8.4)	10 (17.9)	0.042
	AF recurrence within 12 mo. post-blanking, n (%)		91 (18.8)	74 (17.2)	17 (30.4)	0.029
	Re-ablation within 12 mo., n (%)		29 (6)	23 (5.4)	6 (10.7)	0.197
	Rehospitalization within 12 mo., n (%)		105 (21.6)	79 (18.4)	26 (46.4)	< 0.01
		Cardiac cause, n (%)	75 (73.5)	54 (70.1)	21 (84.0)	0.269

sPAP, systolic pulmonary artery pressure; PH, pulmonary hypertension.

* This cohort includes patients with estimated sPAP ≤ 45 mm Hg and those with normal estimated sPAP.

** This cohort includes patients with estimated sPAP > 45 mm Hg.

† Including phrenic nerve palsy/injury, neurologic events, pulmonary events, peripheral vascular events, and pericardial tamponade.

# Including stroke, transient ischemic attack, and persistent phrenic nerve damage.

### 3.4 Impact of pH on AF recurrence following PVI

PVI was successfully achieved in the vast majority of patients (92.6%), with no significant differences observed between the groups (89.3% vs. 93%; p = 0.46). Sinus rhythm at discharge was consistently high across both groups, with an overall rate of 98.4%. (Table 3). Beyond the blanking period, AAD therapy was continued more frequently in patients with high-probability PH (52% vs. 43%).

The high-probability PH group demonstrated a significantly higher rate of post-blanking AF recurrence within one-year (30.4% vs. 17.2%; p = 0.029). Furthermore, the high-probability PH group had a higher 12-month all-cause mortality (3.6% vs. 0.2%; p = 0.037), with all deaths attributed to non-cardiovascular causes, as well as significantly higher rehospitalization rates (46.4% vs. 18.4%; p < 0.01) (Table 3).

**Fig. 1:**
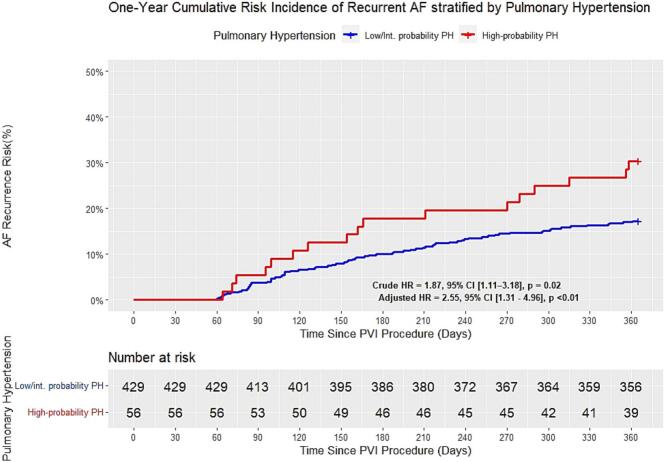
One-Year Cumulative Risk Incidence of Recurrent AF Stratified by PH Probability Following PVI Procedure Kaplan-Meier curves showing one-year cumulative AF recurrence risk after PVI, stratified by high- versus low/intermediate-probability PH. High-probability PH patients (red line, n = 56) demonstrated significantly higher recurrence rates compared to low/intermediate-probability PH patients (blue line, n = 429), with adjusted HR 2.55 (95% CI 1.31–4.96, p < 0.01). **Abbreviation**: AF, atrial fibrillation; CI, confidence interval; Int., intermediate; HR, hazard ratio; PH, pulmonary hypertension; PVI, pulmonary vein isolation. (For interpretation of the references to colour in this figure legend, the reader is referred to the web version of this article.)

Kaplan-Meier analysis demonstrated consistently higher AF recurrence rates in the high-probability PH group (HR: 1.87, 95% CI: 1.1–3.8; p = 0.02) (Fig. 1). After adjusting for key potential confounders, including age, gender, HF, prior IHD, ablation energy source, DM, hypertension (HTN), COPD, renal dysfunction, AF subtype, LVEF, LA size, significant mitral and tricuspid regurgitation, a Cox proportional hazards model identified high-probability PH as an independent predictor of AF recurrence, with a 2.55-fold increased risk (95% CI: 1.31–4.96; p < 0.01) (Fig. 1).

**Fig. 2:**
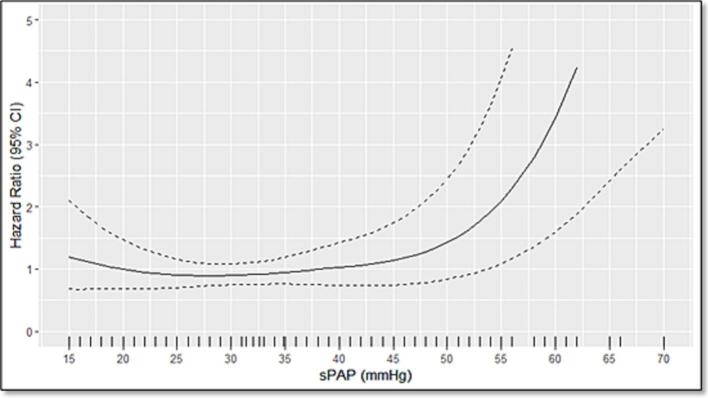
Hazard Ratio for AF Recurrence as a Function of sPAP (mmHg) The solid line represents the estimated hazard ratio, while the dashed lines indicate the 95% CI. Higher sPAP values, particularly above 45 mm Hg, are associated with an increased risk of AF recurrence. **Abbreviation**: AF, atrial fibrillation; sPAP, systolic pulmonary artery pressure; CI, confidence interval.

A multivariable Cox proportional hazards regression analysis using echocardiographic data was performed, modeling sPAP as a continuous variable using non-linear restricted cubic splines. This approach revealed a strong, statistically significant association between sPAP and AF recurrence (p < 0.01), indicating a clear dose–response relationship (Fig. 2).

**Fig. 3:**
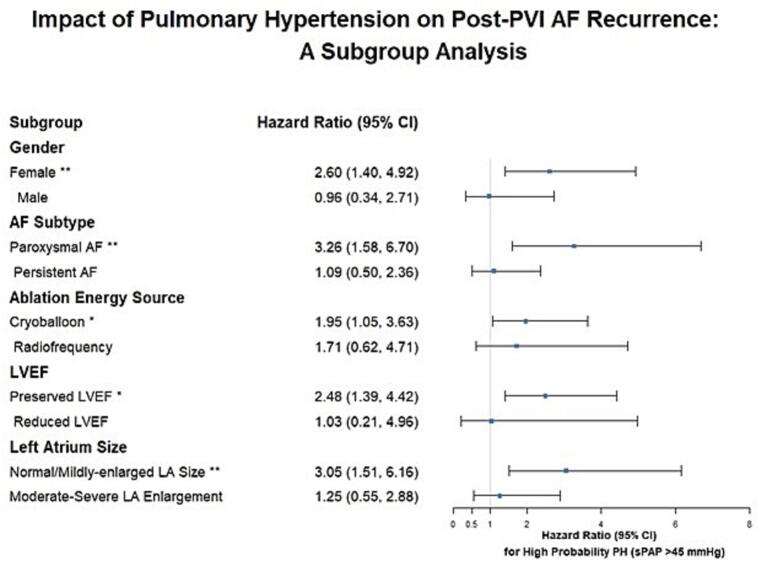
Subgroup Analysis of High-Probability PH on Post-PVI AF Recurrence Subgroup analysis using a Cox proportional hazards model evaluating the impact of high-probability PH (sPAP > 45 mmHg) on AF recurrence following PVI. HR and 95% CI are presented for key subgroups, including gender, AF subtype, ablation energy source, LVEF, and LA size. Subgroups with significant associations are marked (* p < 0.05, ^**^ p < 0.01, ^***^ p < 0.001). **Abbreviation**: AF, atrial fibrillation; CI, confidence interval; HR, hazard ratio; LA, left atrium; LVEF, left ventricular ejection fraction; PH, pulmonary hypertension.

Subgroup analyses using a Cox proportional hazards model revealed that elevated sPAP is significantly associated with AF recurrence across specific subgroups. Among women with high-probability PH, there was a notably higher risk of AF recurrence (HR 2.60, 95% CI: 1.40–4.92, p < 0.01) compared to those with low/intermediate probability or normal sPAP levels. Additionally, higher recurrence risks were observed in patients with paroxysmal AF (HR 3.26, 95% CI: 1.58–6.70, p < 0.01), those undergoing CBA PVI (HR 1.95, 95% CI: 1.05–3.63, p < 0.05), and patients with preserved left ventricular ejection fraction (LVEF) (HR 2.48, 95% CI: 1.39–4.42, p < 0.05). Patients with normal or mildly enlarged LA size also experienced higher recurrence risk (HR 3.05, 95% CI: 1.51–6.16, p < 0.01) (Fig. 3).

## 4 Discussion

This study represents the first large-scale, prospective, multicenter observational analysis investigating the relationship between PH and AF recurrence following PVI, predominantly performed with cryoballoon ablation (CBA). Our findings demonstrate that patients with high-probability PH, indicated by elevated echocardiographic sPAP values, experienced significantly higher AF recurrence rates.

As anticipated, patients with PH demonstrated a distinct baseline profile characterized by a greater burden of comorbidities, including advanced age, higher prevalence of HF, cardiovascular risk factors, and COPD. Suggesting that the association between sPAP and AF recurrence may partly reflect a more advanced underlying disease state rather than PH alone. To account for these baseline differences and enhance the robustness of our findings, we conducted an adjusted multivariable analysis, further confirming our results.

While few previous studies that examined the relationship between PH and post-PVI AF recurrence mainly included participants undergoing PVI with RFA [16], [17], [18], this up-to-date prospective multicenter study predominantly involved patients treated with CBA. Choi et al. explored a single-center, retrospective cohort of 2,379 patients with paroxysmal and persistent AF, finding that elevated sPAP significantly increased the risk of AF recurrence post-ablation [16]. Li et al. performed a retrospective study of 257 patients, utilizing multivariable logistic regression to show that PH was independently associated with AF recurrence [17]. Similarly, Zhang et al. analyzed 300 AF patients retrospectively, demonstrating that increased sPAP was independently linked to post-blanking AF recurrence [18].

A key clinical implication of our findings is that echocardiographic-estimated sPAP can effectively predict the risk of AF recurrence following catheter ablation [1], [16]. Traditional echocardiographic markers, such as anteroposterior LA diameter and the LA volume index (LAVi), have notable drawbacks. While the LA diameter provides a limited view, potentially underestimating the true three-dimensional structure of the atrium, the LAVi is technically complex and highly dependent on operator expertise. In contrast, sPAP emerges as a simpler and more reliable indicator of hemodynamic burden and pulmonary pressures, complementing existing metrics and broadening the scope of pre-procedural assessment [16], [26], [27], [28].

A key consideration relates to the classification of high-probability PH based on echocardiographic sPAP. While RHC remains the gold standard for PH assessment, Doppler echocardiography is a practical and guideline-endorsed method for estimating PH probability, although it may be prone to misclassification, particularly in patients with AF at the time of assessment [9], [19], [22]. Despite the inherent limitations of sPAP estimation, including assumptions regarding right atrial pressure, continuous modeling in our cohort demonstrated a graded association between increasing sPAP and AF recurrence, with a clear risk inflection around 40 mmHg and a steeper increase beyond 45 mmHg, supporting the clinical relevance of this cutoff.

Although acute PVI outcomes were similar across groups, patients with PH experienced higher rates of post-PVI recurrence. This finding may indicate the involvement of non-pulmonary vein triggers, a mechanism particularly relevant in PH where secondary right atrial and ventricular remodeling often occurs [3]. Considering this potential pathophysiological process, and the predominance of CBA in our cohort, a mapping-guided ablation strategy may offer improved efficacy in this specific subpopulation and consider the continuation of anti-arrhythmic medications after PVI. Further studies are warranted to elucidate these mechanisms and validate this hypothesis.

Subgroup analysis performed in this study, while exploratory and limited by reduced statistical power due to sample size imbalance between groups, indicates that the presence of pH is significantly associated with AF recurrence in specific subpopulations, including women, patients with paroxysmal AF, those undergoing CBA, individuals with preserved LVEF, and patients with normal or mildly enlarged LA sizes. This emphasizes the necessity for a personalized surveillance strategy post-PVI, recommending a more thorough follow-up protocol.

A potential clinical implication arising from these findings is whether optimization of pH prior to PVI could influence post-ablation outcomes. In patients with potentially reversible PH, reduction of pulmonary arterial pressure may decrease atrial stretch and hemodynamic burden, analogous to the beneficial effects observed with optimization of modifiable risk factors such as glycemic control, treatment of obstructive sleep apnea, and weight reduction. Conversely, long-standing PH may lead to irreversible atrial and pulmonary vein remodeling, limiting the impact of pH improvement alone on recurrence risk [1], [2]. Accordingly, while targeted PH management may represent a potential adjunctive strategy, its benefit remains theoretical and warrants evaluation in prospective studies.

### 4.1 Limitations

Despite the strengths of this study, which include its up-to-date, prospective, multicenter, and relatively large-scale design, several limitations need to be considered. First, although we adjusted for potential confounders, we cannot completely eliminate the possibility of residual confounding. Second, the use of echocardiographically estimated sPAP, while commonly accepted, might not be as accurate as invasive hemodynamic measurements, posing a risk of misclassification. Third, the small size of the high-probability PH group and the resulting sample imbalance may have limited the power of subgroup analyses. Lastly, the predominance of CBA in our cohort could restrict the applicability of our findings to patients treated with other ablation techniques, such as RFA or pulse field ablation.

## 5 Conclusion

This study emphasizes the importance of pulmonary hypertension, evaluated through echocardiographic sPAP values, in predicting post-PVI AF recurrence. These findings advocate for incorporating sPAP into pre-procedural AF recurrence risk assessments and underscore the need for customized surveillance strategies.

## 6 Ethics and patient consent

Ethical approval was obtained from the ethics committees of all participating institutions, and patients provided written consent.

## CRediT authorship contribution statement

**Eias Massalha:** Writing – review & editing, Writing – original draft, Supervision, Software, Resources, Project administration, Methodology, Investigation, Formal analysis, Data curation, Conceptualization. **Amer Dakka:** Writing – review & editing, Writing – original draft, Validation, Supervision, Methodology, Investigation. **Ibrahim Marai:** Writing – original draft, Data curation. **Yoav Michowitz:** Project administration, Methodology, Data curation, Conceptualization. **Michael Glikson:** Validation, Supervision, Project administration, Data curation. **Yuval Konstantino:** Validation, Project administration, Formal analysis, Conceptualization. **Moti Haim:** Supervision, Project administration, Methodology, Investigation, Conceptualization. **David Luria:** Project administration, Methodology, Data curation. **Alexander Omelchenko:** Resources, Project administration, Investigation, Data curation. **Avishag Laish-Farkash:** Supervision, Project administration, Conceptualization. **Mahmoud Suleiman:** Project administration, Data curation. **Ariel Furer:** Writing – review & editing, Methodology, Data curation. **Eyal Nof:** Writing – review & editing, Project administration, Methodology, Investigation, Data curation. **Roy Beinart:** Writing – review & editing, Writing – original draft, Supervision, Resources, Project administration, Methodology, Investigation, Formal analysis, Data curation, Conceptualization.

## Funding

None.

## Declaration of competing interest

The authors declare that they have no known competing financial interests or personal relationships that could have appeared to influence the work reported in this paper.
